# Tetra­aqua­bis­(2,6-dihy­droxy­benzoato-κ*O* ^1^)(2,6-dihy­droxy­benzoato-κ^2^ *O* ^1^,*O* ^1′^)gadolinium(III) dihydrate

**DOI:** 10.1107/S1600536811016163

**Published:** 2011-05-07

**Authors:** Juangang Wang, Jun Zhang, Tiedan Chen

**Affiliations:** a College of Chemistry and Material Science, Huaibei Normal University, Xiangshan, Huaibei 235000, People’s Republic of China

## Abstract

In the title compound, [Gd(C_7_H_5_O_4_)_3_(H_2_O)_4_]·2H_2_O, the Gd^III^ ion shows a distorted square anti­prismatic coordination formed by four aqua ligands and four O atoms from the three 2,6-dihy­droxy­benzoate (*L*) ligands. Two *L* ligands coordinate the Gd^III^ ion in a monodentate mode, while the third coordinates it in a bidentate–chelating coordination mode. An extensive three-dimensional O—H⋯O hydrogen-bonding network consolidates the crystal packing.

## Related literature

The crystal structures of related complexes with Ho and Tb were reported by Glowiak *et al.* (1999 ▶).
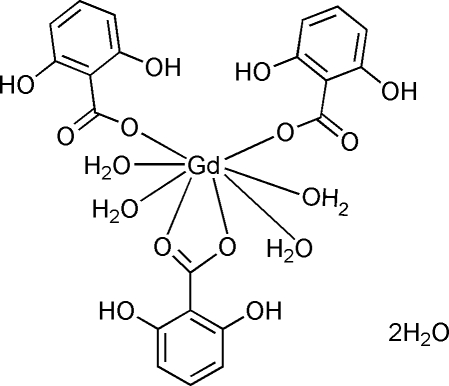


## Experimental

### 

#### Crystal data

[Gd(C_7_H_5_O_4_)_3_(H_2_O)_4_]·2H_2_O
*M* *_r_* = 724.68
Triclinic, 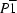

*a* = 10.7666 (4) Å
*b* = 11.3289 (4) Å
*c* = 12.4741 (4) Å
α = 82.270 (1)°
β = 73.066 (1)°
γ = 68.178 (1)°
*V* = 1350.65 (8) Å^3^
*Z* = 2
Mo *K*α radiation
μ = 2.54 mm^−1^
*T* = 294 K
0.24 × 0.20 × 0.18 mm

#### Data collection

Bruker APEXII area-detector diffractometer
Absorption correction: multi-scan (*SADABS*; Bruker, 2007 ▶) *T*_min_ = 0.581, *T*_max_ = 0.658
6854 measured reflections
4691 independent reflections
4301 reflections with *I* > 2σ(*I*)
*R*_int_ = 0.014

#### Refinement

*R*[*F* ^2^ > 2σ(*F* ^2^)] = 0.022
*wR*(*F* ^2^) = 0.052
*S* = 1.04
4691 reflections
415 parameters
H atoms treated by a mixture of independent and constrained refinement
Δρ_max_ = 0.48 e Å^−3^
Δρ_min_ = −0.61 e Å^−3^

### 

Data collection: *SMART* (Bruker, 2007 ▶); cell refinement: *SAINT* (Bruker, 2007 ▶); data reduction: *SAINT*; program(s) used to solve structure: *SHELXS97* (Sheldrick, 2008 ▶); program(s) used to refine structure: *SHELXL97* (Sheldrick, 2008 ▶); molecular graphics: *SHELXTL* (Sheldrick, 2008 ▶); software used to prepare material for publication: *SHELXTL* and *publCIF* (Westrip, 2010 ▶).

## Supplementary Material

Crystal structure: contains datablocks I, global. DOI: 10.1107/S1600536811016163/cv5069sup1.cif

Structure factors: contains datablocks I. DOI: 10.1107/S1600536811016163/cv5069Isup2.hkl

Additional supplementary materials:  crystallographic information; 3D view; checkCIF report

## Figures and Tables

**Table 1:** Hydrogen-bond geometry (Å, °)

*D*—H⋯*A*	*D*—H	H⋯*A*	*D*⋯*A*	*D*—H⋯*A*
O13—H13*A*⋯O17^i^	0.84 (5)	1.88 (5)	2.697 (5)	161 (4)
O14—H14*A*⋯O17^i^	0.77 (5)	1.99 (7)	2.766 (5)	172 (4)
O15—H15*A*⋯O4^ii^	0.83 (5)	1.90 (8)	2.739 (5)	176 (5)
O16—H16*A*⋯O11	0.72 (4)	2.09 (5)	2.735 (5)	151 (2)
O16—H16*A*⋯O12^iii^	0.72 (4)	2.50 (5)	2.865 (5)	113 (7)
O17—H17*A*⋯O18	0.86 (5)	1.81 (5)	2.668 (5)	175 (5)
O18—H18*A*⋯O5^ii^	0.91 (7)	1.88 (5)	2.752 (5)	161 (4)
O13—H13*B*⋯O1^iv^	0.88 (5)	1.94 (5)	2.808 (5)	172 (5)
O14—H14*B*⋯O9^v^	0.84 (5)	1.90 (5)	2.739 (5)	174 (5)
O15—H15*B*⋯O18	0.70 (4)	2.22 (5)	2.917 (5)	176 (6)
O16—H16*B*⋯O2	0.88 (6)	1.89 (5)	2.672 (5)	147 (5)
O17—H17*B*⋯O8^vi^	0.82 (5)	2.03 (5)	2.713 (5)	140 (5)
O18—H18*B*⋯O12^iii^	0.83 (5)	2.08 (5)	2.898 (5)	172 (3)
O1—H1⋯O2	0.82	1.78	2.515 (5)	148
O4—H4⋯O3	0.82	1.82	2.549 (5)	147
O5—H5⋯O6	0.82	1.83	2.566 (5)	148
O8—H8⋯O7	0.82	1.83	2.546 (5)	145
O9—H9⋯O10	0.82	1.82	2.551 (5)	147
O12—H12⋯O11	0.82	1.78	2.512 (5)	148

Symmetry codes: (i) 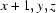; (ii) 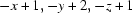; (iii) 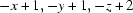; (iv) 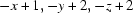; (v) 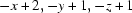; (vi) 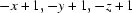.

**Fig. 1.**
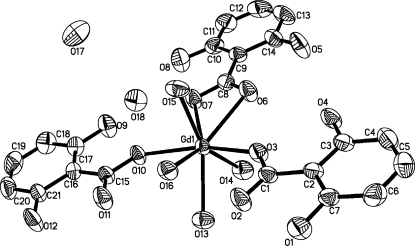
The molecular structure of the title compound, drawn with 50% probability displacement ellipsoids. H atoms omitted for clarity.

## supplementary crystallographic information

### Comment

The title compound is isomorphous with the related Ho and Tb complexes (Glowiak *et al.*, 1999). The coordinating Gd—O bond lengths are in the range 2.344 (2)–2.512 (2) Å. Intermolecular O—H···O hydrogen bonds (Table 1) form an extensive three-dimensional hydrogen-bonding network, which consolidate the crystal packing.

### Experimental

The title complex was synthesized by dissolving 2,6-dihydroxybenzoic acid (10 mmol, 1.54 g) in 25 ml of ethanol. This was followed by the addition of Gadolinium nitrate (3 mmol,1.35 g), dissolved in 20 ml of distilled water. The mixture was stirred at room temperature for 5 h and the resulting white precipitate was filtered, dried and recrystallized from water.

### Refinement

C-bound H atoms were geometrically positioned and refined as riding, with C—H = 0.93 Å and *U*_iso_(H) = 1.2 U_eq_(C). H atoms attached to O atoms were found in a difference Fourier map and isotropically refined.

### Crystal data

**Table:**

[Gd(C_7_H_5_O_4_)_3_(H_2_O)_4_]·2H_2_O	*V* = 1350.65 (8) Å^3^
*M**_r_* = 724.68	*Z* = 2
Triclinic, *P*1	*F*(000) = 722
Hall symbol: -P 1	*D*_x_ = 1.782 Mg m^−^^3^
*a* = 10.7666 (4) Å	Melting point: 476 K
*b* = 11.3289 (4) Å	Mo *K*α radiation, λ = 0.71073 Å
*c* = 12.4741 (4) Å	µ = 2.54 mm^−^^1^
α = 82.270 (1)°	*T* = 294 K
β = 73.066 (1)°	Block, white
γ = 68.178 (1)°	0.24 × 0.20 × 0.18 mm

### Data collection

**Table:**

Bruker APEXII area-detector diffractometer	4691 independent reflections
Radiation source: fine-focus sealed tube	4301 reflections with *I* > 2σ(*I*)
graphite	*R*_int_ = 0.014
ω scans	θ_max_ = 25.0°, θ_min_ = 1.9°
Absorption correction: multi-scan (*SADABS*; Bruker, 2007)	*h* = −12→12
*T*_min_ = 0.581, *T*_max_ = 0.658	*k* = −10→13
6854 measured reflections	*l* = −14→14

### Refinement

**Table:**

Refinement on *F*^2^	Primary atom site location: structure-invariant direct methods
Least-squares matrix: full	Secondary atom site location: difference Fourier map
*R*[*F*^2^ > 2σ(*F*^2^)] = 0.022	Hydrogen site location: inferred from neighbouring sites
*wR*(*F*^2^) = 0.052	H atoms treated by a mixture of independent and constrained refinement
*S* = 1.04	*w* = 1/[σ^2^(*F*_o_^2^) + (0.0247*P*)^2^ + 0.6501*P*] where *P* = (*F*_o_^2^ + 2*F*_c_^2^)/3
4691 reflections	(Δ/σ)_max_ = 0.002
415 parameters	Δρ_max_ = 0.48 e Å^−^^3^
0 restraints	Δρ_min_ = −0.61 e Å^−^^3^

### Special details

**Table:**

Geometry. All e.s.d.'s (except the e.s.d. in the dihedral angle between two l.s. planes) are estimated using the full covariance matrix. The cell e.s.d.'s are taken into account individually in the estimation of e.s.d.'s in distances, angles and torsion angles; correlations between e.s.d.'s in cell parameters are only used when they are defined by crystal symmetry. An approximate (isotropic) treatment of cell e.s.d.'s is used for estimating e.s.d.'s involving l.s. planes.
Refinement. Refinement of *F*^2^ against ALL reflections. The weighted *R*-factor *wR* and goodness of fit *S* are based on *F*^2^, conventional *R*-factors *R* are based on *F*, with *F* set to zero for negative *F*^2^. The threshold expression of *F*^2^ > σ(*F*^2^) is used only for calculating *R*-factors(gt) *etc*. and is not relevant to the choice of reflections for refinement. *R*-factors based on *F*^2^ are statistically about twice as large as those based on *F*, and *R*- factors based on ALL data will be even larger.

### Fractional atomic coordinates and isotropic or equivalent isotropic displacement parameters (Å^2^)

**Table:**

	*x*	*y*	*z*	*U*_iso_*/*U*_eq_	
C1	0.5308 (3)	1.0415 (3)	0.7814 (2)	0.0340 (7)	
C2	0.4935 (3)	1.1804 (3)	0.7630 (2)	0.0332 (7)	
C3	0.5284 (3)	1.2357 (3)	0.6563 (3)	0.0377 (8)	
C4	0.4932 (4)	1.3661 (3)	0.6407 (3)	0.0508 (9)	
H4A	0.5164	1.4018	0.5695	0.061*	
C5	0.4231 (4)	1.4428 (3)	0.7326 (3)	0.0542 (10)	
H5A	0.4005	1.5304	0.7225	0.065*	
C6	0.3861 (4)	1.3924 (3)	0.8387 (3)	0.0477 (9)	
H6	0.3384	1.4454	0.8995	0.057*	
C7	0.4203 (3)	1.2626 (3)	0.8539 (3)	0.0365 (7)	
C8	0.7815 (4)	0.7434 (3)	0.4254 (2)	0.0365 (7)	
C9	0.8391 (3)	0.7349 (3)	0.3029 (3)	0.0370 (7)	
C10	0.9056 (4)	0.6168 (3)	0.2523 (3)	0.0452 (9)	
C11	0.9649 (4)	0.6091 (4)	0.1376 (3)	0.0570 (10)	
H11	1.0097	0.5304	0.1042	0.068*	
C12	0.9566 (5)	0.7183 (5)	0.0745 (3)	0.0635 (12)	
H12A	0.9973	0.7125	−0.0022	0.076*	
C13	0.8907 (4)	0.8359 (4)	0.1199 (3)	0.0597 (11)	
H13	0.8856	0.9089	0.0746	0.072*	
C14	0.8314 (4)	0.8445 (3)	0.2349 (3)	0.0455 (9)	
C15	0.7184 (3)	0.4653 (3)	0.7986 (2)	0.0350 (7)	
C16	0.7694 (3)	0.3260 (3)	0.7872 (3)	0.0332 (7)	
C17	0.8403 (4)	0.2674 (3)	0.6841 (3)	0.0403 (8)	
C18	0.8838 (4)	0.1373 (3)	0.6746 (3)	0.0540 (10)	
H18	0.9310	0.0998	0.6055	0.065*	
C19	0.8556 (4)	0.0642 (4)	0.7698 (4)	0.0604 (11)	
H19	0.8838	−0.0234	0.7640	0.072*	
C20	0.7869 (4)	0.1174 (4)	0.8733 (4)	0.0564 (10)	
H20	0.7691	0.0661	0.9364	0.068*	
C21	0.7448 (4)	0.2473 (3)	0.8828 (3)	0.0404 (8)	
Gd1	0.702869 (16)	0.750791 (13)	0.665777 (11)	0.02894 (6)	
H13A	0.877 (5)	0.730 (4)	0.821 (4)	0.069 (15)*	
H14A	0.971 (5)	0.755 (5)	0.642 (4)	0.079 (18)*	
H15A	0.467 (4)	0.786 (4)	0.568 (4)	0.062 (13)*	
H16A	0.535 (4)	0.704 (4)	0.867 (3)	0.046 (14)*	
H17A	0.122 (5)	0.744 (4)	0.776 (4)	0.085 (17)*	
H18A	0.228 (7)	0.883 (7)	0.793 (6)	0.15 (3)*	
H13B	0.745 (5)	0.750 (4)	0.894 (4)	0.087 (16)*	
H14B	0.991 (5)	0.729 (4)	0.543 (4)	0.087 (17)*	
H15B	0.437 (5)	0.772 (4)	0.668 (4)	0.059 (16)*	
H16B	0.494 (6)	0.831 (5)	0.872 (5)	0.11 (2)*	
H17B	0.108 (5)	0.637 (5)	0.750 (4)	0.081 (17)*	
H18B	0.265 (5)	0.779 (4)	0.868 (4)	0.081 (17)*	
O1	0.3847 (3)	1.2146 (2)	0.95957 (18)	0.0484 (6)	
H1	0.4128	1.1367	0.9584	0.073*	
O2	0.4931 (3)	0.9972 (2)	0.87718 (18)	0.0518 (7)	
O3	0.6016 (2)	0.96988 (19)	0.69634 (16)	0.0388 (5)	
O4	0.5976 (3)	1.1625 (2)	0.56387 (17)	0.0483 (6)	
H4	0.6186	1.0871	0.5833	0.072*	
O5	0.7661 (3)	0.9615 (2)	0.2789 (2)	0.0672 (8)	
H5	0.7339	0.9546	0.3466	0.101*	
O6	0.7188 (3)	0.8501 (2)	0.47171 (18)	0.0468 (6)	
O7	0.7986 (3)	0.6422 (2)	0.48681 (17)	0.0439 (6)	
O8	0.9160 (3)	0.5071 (2)	0.3133 (2)	0.0652 (8)	
H8	0.8706	0.5233	0.3783	0.098*	
O9	0.8689 (3)	0.3387 (2)	0.58862 (18)	0.0538 (7)	
H9	0.8325	0.4144	0.6029	0.081*	
O10	0.7447 (2)	0.53404 (19)	0.71024 (16)	0.0380 (5)	
O11	0.6501 (3)	0.5131 (2)	0.89201 (18)	0.0520 (7)	
O12	0.6791 (3)	0.2971 (2)	0.98503 (19)	0.0577 (7)	
H12	0.6592	0.3746	0.9799	0.087*	
O13	0.7942 (3)	0.7430 (2)	0.8236 (2)	0.0445 (6)	
O14	0.9283 (3)	0.7671 (3)	0.5988 (2)	0.0478 (6)	
O15	0.4977 (3)	0.7588 (3)	0.6240 (3)	0.0486 (7)	
O16	0.5253 (3)	0.7617 (3)	0.8331 (2)	0.0411 (6)	
O17	0.0657 (3)	0.7124 (3)	0.7653 (2)	0.0562 (7)	
O18	0.2460 (3)	0.7987 (3)	0.8066 (3)	0.0568 (7)	

### Atomic displacement parameters (Å^2^)

**Table:**

	*U*^11^	*U*^22^	*U*^33^	*U*^12^	*U*^13^	*U*^23^
C1	0.0415 (19)	0.0323 (17)	0.0258 (16)	−0.0083 (14)	−0.0103 (14)	−0.0029 (13)
C2	0.0367 (18)	0.0332 (17)	0.0284 (16)	−0.0069 (14)	−0.0130 (13)	−0.0023 (13)
C3	0.042 (2)	0.0387 (18)	0.0308 (17)	−0.0090 (15)	−0.0138 (14)	0.0003 (14)
C4	0.067 (3)	0.041 (2)	0.042 (2)	−0.0171 (19)	−0.0187 (18)	0.0097 (16)
C5	0.064 (3)	0.0315 (19)	0.063 (3)	−0.0087 (18)	−0.022 (2)	0.0003 (17)
C6	0.052 (2)	0.038 (2)	0.049 (2)	−0.0056 (17)	−0.0154 (18)	−0.0134 (16)
C7	0.042 (2)	0.0345 (18)	0.0318 (17)	−0.0081 (15)	−0.0135 (14)	−0.0057 (13)
C8	0.045 (2)	0.0417 (19)	0.0239 (15)	−0.0192 (16)	−0.0054 (14)	−0.0017 (14)
C9	0.0374 (19)	0.050 (2)	0.0244 (15)	−0.0190 (16)	−0.0058 (13)	0.0009 (14)
C10	0.054 (2)	0.056 (2)	0.0294 (17)	−0.0244 (19)	−0.0073 (16)	−0.0090 (16)
C11	0.068 (3)	0.070 (3)	0.0319 (19)	−0.027 (2)	−0.0029 (18)	−0.0148 (19)
C12	0.065 (3)	0.105 (4)	0.0235 (18)	−0.037 (3)	−0.0052 (18)	−0.005 (2)
C13	0.063 (3)	0.081 (3)	0.0317 (19)	−0.030 (2)	−0.0108 (18)	0.020 (2)
C14	0.052 (2)	0.050 (2)	0.0336 (18)	−0.0190 (18)	−0.0118 (16)	0.0075 (16)
C15	0.0389 (19)	0.0349 (17)	0.0276 (16)	−0.0133 (14)	−0.0046 (14)	0.0025 (13)
C16	0.0327 (18)	0.0315 (17)	0.0342 (17)	−0.0127 (14)	−0.0059 (14)	0.0010 (13)
C17	0.0373 (19)	0.0349 (18)	0.0434 (19)	−0.0106 (15)	−0.0044 (15)	−0.0038 (15)
C18	0.052 (2)	0.041 (2)	0.059 (2)	−0.0087 (18)	−0.0056 (19)	−0.0128 (18)
C19	0.063 (3)	0.0304 (19)	0.088 (3)	−0.0133 (19)	−0.024 (2)	−0.002 (2)
C20	0.062 (3)	0.042 (2)	0.069 (3)	−0.0244 (19)	−0.026 (2)	0.023 (2)
C21	0.046 (2)	0.042 (2)	0.0365 (18)	−0.0207 (16)	−0.0128 (16)	0.0112 (15)
Gd1	0.03710 (10)	0.02723 (9)	0.01941 (8)	−0.01211 (7)	−0.00154 (6)	−0.00127 (6)
O1	0.0634 (17)	0.0415 (14)	0.0304 (12)	−0.0094 (13)	−0.0058 (11)	−0.0097 (10)
O2	0.083 (2)	0.0354 (13)	0.0248 (12)	−0.0148 (13)	−0.0049 (12)	−0.0002 (10)
O3	0.0524 (15)	0.0315 (12)	0.0253 (11)	−0.0092 (10)	−0.0056 (10)	−0.0034 (9)
O4	0.0705 (18)	0.0412 (14)	0.0250 (11)	−0.0128 (13)	−0.0109 (11)	0.0023 (10)
O5	0.089 (2)	0.0483 (16)	0.0426 (15)	−0.0147 (15)	−0.0049 (15)	0.0146 (12)
O6	0.0646 (17)	0.0373 (13)	0.0286 (12)	−0.0137 (12)	−0.0031 (11)	−0.0006 (10)
O7	0.0685 (17)	0.0356 (13)	0.0244 (11)	−0.0211 (12)	−0.0041 (11)	0.0006 (10)
O8	0.104 (3)	0.0460 (16)	0.0382 (14)	−0.0290 (16)	−0.0009 (15)	−0.0107 (12)
O9	0.0708 (19)	0.0442 (14)	0.0305 (12)	−0.0174 (14)	0.0089 (12)	−0.0068 (11)
O10	0.0517 (14)	0.0314 (12)	0.0242 (11)	−0.0150 (10)	−0.0007 (10)	0.0020 (9)
O11	0.0757 (19)	0.0404 (14)	0.0274 (12)	−0.0186 (13)	0.0032 (12)	−0.0027 (10)
O12	0.078 (2)	0.0539 (16)	0.0328 (13)	−0.0260 (15)	−0.0044 (13)	0.0136 (11)
O13	0.0400 (15)	0.0621 (17)	0.0298 (13)	−0.0185 (13)	−0.0047 (11)	−0.0047 (11)
O14	0.0431 (16)	0.0630 (18)	0.0321 (14)	−0.0193 (13)	0.0007 (13)	−0.0057 (13)
O15	0.0488 (18)	0.0697 (19)	0.0293 (14)	−0.0218 (14)	−0.0128 (14)	0.0000 (13)
O16	0.0433 (15)	0.0522 (17)	0.0260 (12)	−0.0206 (13)	−0.0019 (10)	0.0002 (13)
O17	0.0467 (17)	0.0580 (19)	0.0679 (19)	−0.0180 (15)	−0.0125 (14)	−0.0215 (15)
O18	0.0658 (19)	0.0581 (19)	0.0603 (19)	−0.0311 (15)	−0.0308 (15)	0.0126 (15)

### Geometric parameters (Å, °)

**Table:**

C1—O2	1.245 (4)	C17—C18	1.381 (5)
C1—O3	1.290 (3)	C18—C19	1.378 (5)
C1—C2	1.475 (4)	C18—H18	0.9300
C2—C3	1.402 (4)	C19—C20	1.378 (6)
C2—C7	1.412 (4)	C19—H19	0.9300
C3—O4	1.367 (4)	C20—C21	1.380 (5)
C3—C4	1.383 (4)	C20—H20	0.9300
C4—C5	1.384 (5)	C21—O12	1.348 (4)
C4—H4A	0.9300	Gd1—O3	2.344 (2)
C5—C6	1.377 (5)	Gd1—O10	2.345 (2)
C5—H5A	0.9300	Gd1—O16	2.366 (2)
C6—C7	1.377 (4)	Gd1—O15	2.380 (3)
C6—H6	0.9300	Gd1—O14	2.394 (3)
C7—O1	1.360 (4)	Gd1—O13	2.422 (2)
C8—O6	1.267 (4)	Gd1—O7	2.453 (2)
C8—O7	1.275 (4)	Gd1—O6	2.515 (2)
C8—C9	1.474 (4)	O1—H1	0.8200
C9—C14	1.396 (5)	O4—H4	0.8200
C9—C10	1.398 (5)	O5—H5	0.8200
C10—O8	1.351 (4)	O8—H8	0.8200
C10—C11	1.386 (5)	O9—H9	0.8200
C11—C12	1.362 (6)	O12—H12	0.8200
C11—H11	0.9300	O13—H13A	0.84 (5)
C12—C13	1.365 (6)	O13—H13B	0.88 (5)
C12—H12A	0.9300	O14—H14A	0.77 (5)
C13—C14	1.390 (5)	O14—H14B	0.84 (5)
C13—H13	0.9300	O15—H15A	0.83 (5)
C14—O5	1.353 (4)	O15—H15B	0.70 (4)
C15—O11	1.251 (4)	O16—H16A	0.72 (4)
C15—O10	1.286 (3)	O16—H16B	0.88 (6)
C15—C16	1.478 (4)	O17—H17A	0.86 (5)
C16—C17	1.399 (4)	O17—H17B	0.82 (5)
C16—C21	1.411 (4)	O18—H18A	0.91 (7)
C17—O9	1.370 (4)	O18—H18B	0.83 (5)
			
O2—C1—O3	122.3 (3)	C19—C20—H20	120.2
O2—C1—C2	119.6 (3)	C21—C20—H20	120.2
O3—C1—C2	118.1 (3)	O12—C21—C20	118.5 (3)
C3—C2—C7	117.6 (3)	O12—C21—C16	120.9 (3)
C3—C2—C1	122.1 (3)	C20—C21—C16	120.6 (3)
C7—C2—C1	120.4 (3)	O3—Gd1—O10	156.25 (7)
O4—C3—C4	117.8 (3)	O3—Gd1—O16	77.50 (10)
O4—C3—C2	121.0 (3)	O10—Gd1—O16	79.15 (10)
C4—C3—C2	121.2 (3)	O3—Gd1—O15	88.85 (9)
C3—C4—C5	119.1 (3)	O10—Gd1—O15	87.16 (9)
C3—C4—H4A	120.4	O16—Gd1—O15	69.80 (10)
C5—C4—H4A	120.4	O3—Gd1—O14	90.77 (9)
C6—C5—C4	121.5 (3)	O10—Gd1—O14	104.82 (9)
C6—C5—H5A	119.2	O16—Gd1—O14	141.00 (10)
C4—C5—H5A	119.2	O15—Gd1—O14	147.97 (11)
C5—C6—C7	119.3 (3)	O3—Gd1—O13	84.56 (8)
C5—C6—H6	120.4	O10—Gd1—O13	83.92 (8)
C7—C6—H6	120.4	O16—Gd1—O13	71.30 (9)
O1—C7—C6	118.4 (3)	O15—Gd1—O13	141.05 (10)
O1—C7—C2	120.3 (3)	O14—Gd1—O13	70.64 (10)
C6—C7—C2	121.3 (3)	O3—Gd1—O7	128.31 (7)
O6—C8—O7	118.9 (3)	O10—Gd1—O7	73.64 (7)
O6—C8—C9	121.2 (3)	O16—Gd1—O7	138.91 (9)
O7—C8—C9	119.8 (3)	O15—Gd1—O7	78.57 (10)
C14—C9—C10	118.4 (3)	O14—Gd1—O7	76.67 (9)
C14—C9—C8	120.8 (3)	O13—Gd1—O7	133.70 (9)
C10—C9—C8	120.8 (3)	O3—Gd1—O6	76.04 (7)
O8—C10—C11	118.1 (3)	O10—Gd1—O6	125.25 (7)
O8—C10—C9	121.4 (3)	O16—Gd1—O6	136.37 (9)
C11—C10—C9	120.5 (3)	O15—Gd1—O6	75.59 (10)
C12—C11—C10	119.2 (4)	O14—Gd1—O6	73.26 (9)
C12—C11—H11	120.4	O13—Gd1—O6	138.47 (9)
C10—C11—H11	120.4	O7—Gd1—O6	52.28 (7)
C11—C12—C13	122.4 (3)	C7—O1—H1	109.5
C11—C12—H12A	118.8	C1—O3—Gd1	136.40 (19)
C13—C12—H12A	118.8	C3—O4—H4	109.5
C12—C13—C14	118.8 (4)	C14—O5—H5	109.5
C12—C13—H13	120.6	C8—O6—Gd1	92.90 (18)
C14—C13—H13	120.6	C8—O7—Gd1	95.61 (18)
O5—C14—C13	118.3 (3)	C10—O8—H8	109.5
O5—C14—C9	121.0 (3)	C17—O9—H9	109.5
C13—C14—C9	120.6 (4)	C15—O10—Gd1	136.89 (19)
O11—C15—O10	122.0 (3)	C21—O12—H12	109.5
O11—C15—C16	120.0 (3)	Gd1—O13—H13A	126 (3)
O10—C15—C16	118.0 (3)	Gd1—O13—H13B	124 (3)
C17—C16—C21	117.8 (3)	H13A—O13—H13B	110 (4)
C17—C16—C15	122.6 (3)	Gd1—O14—H14A	118 (4)
C21—C16—C15	119.6 (3)	Gd1—O14—H14B	126 (3)
O9—C17—C18	117.8 (3)	H14A—O14—H14B	100 (5)
O9—C17—C16	120.4 (3)	Gd1—O15—H15A	132 (3)
C18—C17—C16	121.8 (3)	Gd1—O15—H15B	118 (4)
C19—C18—C17	118.6 (4)	H15A—O15—H15B	102 (5)
C19—C18—H18	120.7	Gd1—O16—H16A	115 (3)
C17—C18—H18	120.7	Gd1—O16—H16B	117 (4)
C18—C19—C20	121.8 (3)	H16A—O16—H16B	114 (5)
C18—C19—H19	119.1	H17A—O17—H17B	109 (5)
C20—C19—H19	119.1	H18A—O18—H18B	109 (5)
C19—C20—C21	119.5 (3)		
			
O2—C1—C2—C3	−177.4 (3)	C16—C17—C18—C19	0.1 (6)
O3—C1—C2—C3	2.3 (5)	C17—C18—C19—C20	−0.6 (6)
O2—C1—C2—C7	2.5 (5)	C18—C19—C20—C21	0.0 (6)
O3—C1—C2—C7	−177.8 (3)	C19—C20—C21—O12	−179.3 (4)
C7—C2—C3—O4	−179.1 (3)	C19—C20—C21—C16	1.1 (6)
C1—C2—C3—O4	0.8 (5)	C17—C16—C21—O12	178.9 (3)
C7—C2—C3—C4	0.5 (5)	C15—C16—C21—O12	−2.1 (5)
C1—C2—C3—C4	−179.6 (3)	C17—C16—C21—C20	−1.5 (5)
O4—C3—C4—C5	−180.0 (3)	C15—C16—C21—C20	177.6 (3)
C2—C3—C4—C5	0.4 (5)	O2—C1—O3—Gd1	−4.0 (5)
C3—C4—C5—C6	−0.9 (6)	C2—C1—O3—Gd1	176.3 (2)
C4—C5—C6—C7	0.4 (6)	O10—Gd1—O3—C1	13.5 (4)
C5—C6—C7—O1	179.0 (3)	O16—Gd1—O3—C1	24.3 (3)
C5—C6—C7—C2	0.5 (5)	O15—Gd1—O3—C1	93.8 (3)
C3—C2—C7—O1	−179.4 (3)	O14—Gd1—O3—C1	−118.2 (3)
C1—C2—C7—O1	0.7 (5)	O13—Gd1—O3—C1	−47.8 (3)
C3—C2—C7—C6	−0.9 (5)	O7—Gd1—O3—C1	168.2 (3)
C1—C2—C7—C6	179.2 (3)	O6—Gd1—O3—C1	169.2 (3)
O6—C8—C9—C14	2.9 (5)	O7—C8—O6—Gd1	5.2 (3)
O7—C8—C9—C14	−174.8 (3)	C9—C8—O6—Gd1	−172.5 (3)
O6—C8—C9—C10	−178.7 (3)	O3—Gd1—O6—C8	178.0 (2)
O7—C8—C9—C10	3.7 (5)	O10—Gd1—O6—C8	−13.7 (2)
C14—C9—C10—O8	−179.8 (3)	O16—Gd1—O6—C8	−127.7 (2)
C8—C9—C10—O8	1.8 (5)	O15—Gd1—O6—C8	−89.5 (2)
C14—C9—C10—C11	1.3 (5)	O14—Gd1—O6—C8	82.9 (2)
C8—C9—C10—C11	−177.1 (3)	O13—Gd1—O6—C8	113.4 (2)
O8—C10—C11—C12	−179.5 (4)	O7—Gd1—O6—C8	−2.98 (18)
C9—C10—C11—C12	−0.5 (6)	O6—C8—O7—Gd1	−5.3 (3)
C10—C11—C12—C13	−0.6 (6)	C9—C8—O7—Gd1	172.4 (3)
C11—C12—C13—C14	0.9 (7)	O3—Gd1—O7—C8	4.2 (2)
C12—C13—C14—O5	−179.9 (4)	O10—Gd1—O7—C8	173.9 (2)
C12—C13—C14—C9	0.0 (6)	O16—Gd1—O7—C8	123.3 (2)
C10—C9—C14—O5	178.8 (3)	O15—Gd1—O7—C8	83.5 (2)
C8—C9—C14—O5	−2.8 (5)	O14—Gd1—O7—C8	−76.0 (2)
C10—C9—C14—C13	−1.1 (5)	O13—Gd1—O7—C8	−121.8 (2)
C8—C9—C14—C13	177.4 (3)	O6—Gd1—O7—C8	2.98 (19)
O11—C15—C16—C17	176.8 (3)	O11—C15—O10—Gd1	2.3 (5)
O10—C15—C16—C17	−2.2 (5)	C16—C15—O10—Gd1	−178.7 (2)
O11—C15—C16—C21	−2.3 (5)	O3—Gd1—O10—C15	−12.1 (4)
O10—C15—C16—C21	178.8 (3)	O16—Gd1—O10—C15	−22.7 (3)
C21—C16—C17—O9	−179.1 (3)	O15—Gd1—O10—C15	−92.7 (3)
C15—C16—C17—O9	1.8 (5)	O14—Gd1—O10—C15	117.4 (3)
C21—C16—C17—C18	0.9 (5)	O13—Gd1—O10—C15	49.3 (3)
C15—C16—C17—C18	−178.2 (3)	O7—Gd1—O10—C15	−171.6 (3)
O9—C17—C18—C19	−179.9 (4)	O6—Gd1—O10—C15	−162.8 (3)

### Hydrogen-bond geometry (Å, °)

**Table:**

*D*—H···*A*	*D*—H	H···*A*	*D*···*A*	*D*—H···*A*
O13—H13A···O17^i^	0.84 (5)	1.88 (5)	2.697 (5)	161 (4)
O14—H14A···O17^i^	0.77 (5)	1.99 (7)	2.766 (5)	172 (4)
O15—H15A···O4^ii^	0.83 (5)	1.90 (8)	2.739 (5)	176 (5)
O16—H16A···O11	0.72 (4)	2.09 (5)	2.735 (5)	151 (2)
O16—H16A···O12^iii^	0.72 (4)	2.50 (5)	2.865 (5)	113 (7)
O17—H17A···O18	0.86 (5)	1.81 (5)	2.668 (5)	175 (5)
O18—H18A···O5^ii^	0.91 (7)	1.88 (5)	2.752 (5)	161 (4)
O13—H13B···O1^iv^	0.88 (5)	1.94 (5)	2.808 (5)	172 (5)
O14—H14B···O9^v^	0.84 (5)	1.90 (5)	2.739 (5)	174 (5)
O15—H15B···O18	0.70 (4)	2.22 (5)	2.917 (5)	176 (6)
O16—H16B···O2	0.88 (6)	1.89 (5)	2.672 (5)	147 (5)
O17—H17B···O8^vi^	0.82 (5)	2.03 (5)	2.713 (5)	140 (5)
O18—H18B···O12^iii^	0.83 (5)	2.08 (5)	2.898 (5)	172 (3)
O1—H1···O2	0.82	1.78	2.515 (5)	148
O4—H4···O3	0.82	1.82	2.549 (5)	147
O5—H5···O6	0.82	1.83	2.566 (5)	148
O8—H8···O7	0.82	1.83	2.546 (5)	145
O9—H9···O10	0.82	1.82	2.551 (5)	147
O12—H12···O11	0.82	1.78	2.512 (5)	148

Symmetry codes: (i) *x*+1, *y*, *z*; (ii) −*x*+1, −*y*+2, −*z*+1; (iii) −*x*+1, −*y*+1, −*z*+2; (iv) −*x*+1, −*y*+2, −*z*+2; (v) −*x*+2, −*y*+1, −*z*+1; (vi) −*x*+1, −*y*+1, −*z*+1.
